# Biphenotypic Sinonasal Sarcoma: Literature Review of a Peculiar Pathological Entity—The Neurosurgical Point of View

**DOI:** 10.3390/cancers16223747

**Published:** 2024-11-06

**Authors:** Sergio Corvino, Giuseppe Corazzelli, Giuseppe Mariniello, Adriana Iuliano, Roberto Altieri, Giuseppe Pontillo, Diego Strianese, Manlio Barbarisi, Andrea Elefante, Oreste de Divitiis

**Affiliations:** 1 Department of Neurosciences and Reproductive and Odontostomatological Sciences, Division of Neurosurgery, University of Naples “Federico II”, 80131 Naples, Italy; giucoraz@gmail.com (G.C.); giumarin@unina.it (G.M.); oreste.dedivitiis@unina.it (O.d.D.); 2 Department of Neurosciences and Reproductive and Odontostomatological Sciences, Division of Ophthalmology, University of Naples “Federico II”, 80131 Naples, Italy; adrianaiuliano@yahoo.it (A.I.); diego.strianese@unina.it (D.S.); 3 Multidisciplinary Department of Medical-Surgical and Dental Specialties, University of Campania “Luigi Vanvitelli”, 81100 Caserta, Italy; roberto.altieri.87@gmail.com (R.A.); manlio.barbarisi@unicampania.it (M.B.); 4 Department of Advanced Biomedical Sciences, University of Naples “Federico II”, 80131 Naples, Italy; giuseppe.pontillo@unina.it (G.P.); aelefant@unina.it (A.E.)

**Keywords:** biphenotypic sarcoma, endoscopy, endoscopic endonasal approach, transorbital approach, paranasal sinus tumors

## Abstract

Background: Biphenotypic sinonasal sarcoma (BSNS) is a low-grade tumor of the sinonasal tract with frequent extension to the orbit and skull base. Due to its rare incidence and recent histopathological and molecular characterization, little data are available in regard to its natural history, treatment and surveillance protocol. Methods: A comprehensive literature review in Embase online electronic databases on BSNS was made. The analyzed factors included the patients’ sex and age, presenting symptoms and signs, anatomical origin and pattern of growth of the tumor, immunohistochemical and molecular features, time to treatment, type of treatment, surgical approach, extent of resection, peri- and post-operative complications, adjuvant therapies, clinical outcome, recurrence and overall survival rates. Results: This literature review involved 34 studies for an overall series of 149 cases of BSNS. The female (66.9%) and middle-aged populations (median 54.88 years old) were mainly affected. The most frequent clinical onset was nasal obstruction (81%), followed by facial discomfort (44%), epistaxis (15.5%) and ocular impairment (14.3%). Ethmoid sinus (67.8%) and nasal cavity (45.4%) were the most common anatomical site of tumor origin, while an extension to the orbit and skull base was registered in 28.7% and 24.5% of cases. Surgery was the main treatment, especially in the form of endoscopic endonasal approach (56.9%), and allowed for gross total resection in 79% of cases. The recurrence rate was 26.2%; three cases of tumor-related death were reported. Median follow-up was 4.6 years. Conclusions: Biphenotypic sinonasal sarcoma is a rare and unique tumoral entity in terms of biological and clinical behavior. Based on the current knowledge, surgery plays the leading role in treatment, accounting for gross total tumor resection in most cases, allowing for clinical symptom and sign resolution and presenting a low rate of perioperative complications. The type of approach and the aim of surgery should be assessed case by case according to patient and pathology features and the surgeon’s experience, as well as the aim of the treatment. Further studies including large surgical series and with long follow-up are required to define prognostic factors and guidelines of treatment for this peculiar pathological entity.

## Simple Summary

Biphenotypic sinonasal sarcoma is a rare low-grade tumor arising from the mucosa of the upper respiratory tract characterized by aggressive biological behavior and a high tendency to invade the skull base and the orbit. The main presenting symptoms and signs include nasal obstruction, facial discomfort, epistaxis and ocular impairment. Surgical resection represents the gold standard of treatment, allowing for the resolution of clinical manifestations and gross total tumor resection in most cases; the role of adjuvant treatments is unclear. The local recurrence rate after treatment is 26%. Tumor-related mortality is very rare.

## 1. Introduction

Biphenotypic sinonasal sarcoma (BSNS), first described in 2012 by Lewis et al. [1] as a “low-grade sinonasal sarcoma with neural and myogenic differentiation” and introduced in the fourth WHO classification of head and neck tumors in 2017 [2], is a rare low-grade tumor of the sinonasal tract [3], with its origin in the paranasal sinuses or nasal cavity, with a tendency to invade the orbit and/or skull base.

The “biphenotypic” aspect of the tumor is due to the coexistence of both neural and myogenic markers in immunophenotypic studies. Indeed, the tumor exhibits immunoreactivity for both S100 (neural marker) and smooth muscle markers (smooth muscle actin (SMA), muscle specific actin (MSA) or calponin); nevertheless, the distribution of the intensity and the extent of staining are variable. Several sinonasal diseases, such as schwannoma, fibrosarcoma, leiomyosarcoma, malignant peripheral nerve sheath tumor, solitary fibrosus tumor, and fibromatosis, can mimic the BSNS from a histopathological point of view. Thus, a proper diagnosis requires immunophenotyping and immunofluorescence studies [4].

Due to the rarity of this pathological entity and its recent histopathological and molecular characterization, studies including a long follow-up and large surgical series are lacking; thus, nowadays, it is difficult to identify the main prognostic factors, as well as to provide well-defined guidelines of treatment and surveillance protocol. Therefore, the aim of the present study was to retrospectively analyze, from a detailed and comprehensive literature review on BSNS, the demographic, clinical, radiological and pathological features, as well as treatment and outcome, of this rare tumor entity.

## 2. Methods

**Figure 1:**
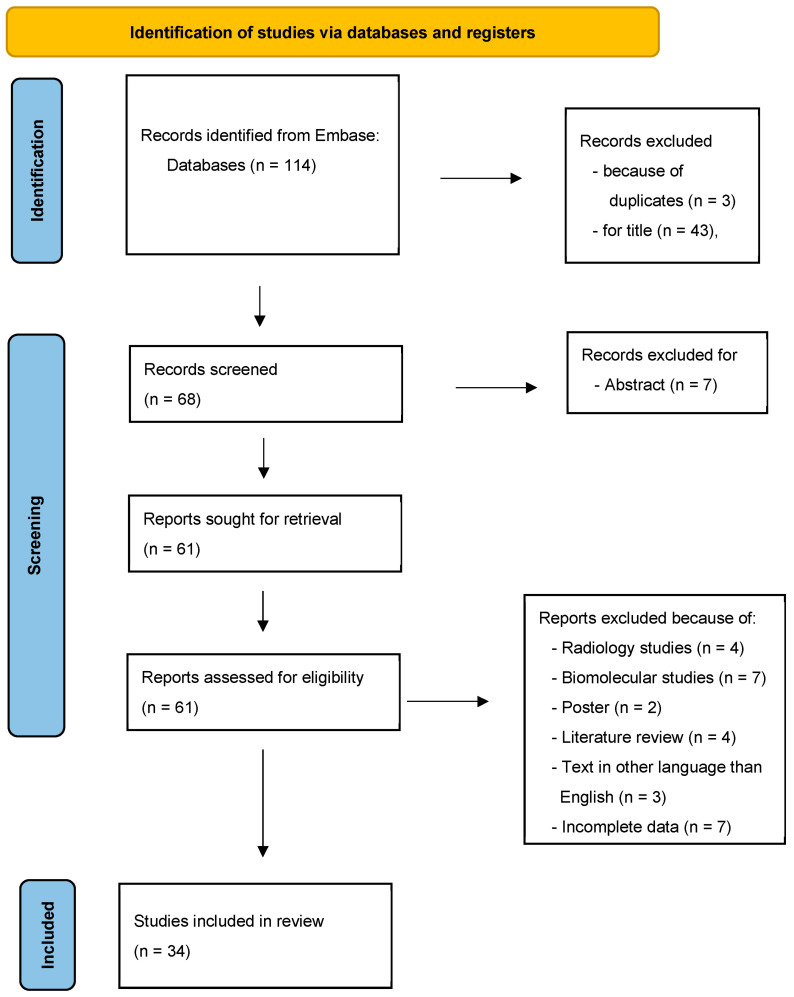
Flowchart showing the methods for the selection of the studies included in this review. From [5].

A Medline search up from January 2012 to May 2024 in the Embase online electronic database was conducted in accordance with the Preferred Reporting Items for Systematic Reviews and Meta-Analysis (PRISMA) guidelines [5], by using the following key phrases: “biphenotypic sinonasal sarcoma” OR “low-grade sinonasal sarcoma”, “biphenotypic sarcoma”, “frontal sinus”, “ethmoid sinus”, “maxillary sinus”, “orbit”. They were combined as follows: (“biphenotypic sinonasal sarcoma” AND “orbit”), (“frontal sinus” AND “biphenotypic sarcoma”), (“ethmoid sinus” AND “biphenotypic sarcoma”), (“maxillary sinus” AND “biphenotypic sarcoma”) (“frontal sinus” AND “ethmoid sinus” AND “maxillary sinus” AND “biphenotypic sarcoma”), (“frontal sinus” AND “ethmoid sinus” AND “orbit” AND “biphenotypic sarcoma”). After duplicate removal, all abstracts were evaluated, and each article of interest was marked for further review. The full text of the marked studies was independently screened by two authors (S.C. and G.C.) and included in this systematic review following the inclusion and exclusion criteria, as summarized in Figure 1.

The inclusion criteria were surgical series, reviews, and case reports in the English language concerning BSNS cases with immunohistochemical diagnosis confirmed or not by molecular exams, and studies reporting clinical and surgical data. Demographic (sex and age), clinical (presenting symptoms), radiological, (anatomical origin, skull base and orbital involvement), pathological (immunohistochemical and molecular diagnosis), treatment (time to treatment, type of treatment, surgical approach, extent of resection, complications) and outcome (clinical, recurrence, overall survival) data were analyzed.

### Statistical Analysis

The normality Shapiro–Wilk test was adopted for categorical and qualitative analyses, *p* values lower than 0.05 were considered statistically significant.

## 3. Results

A comprehensive systematic literature review disclosed 114 studies. After duplicate removal, and screening of the full texts of the marked studies included according to the inclusion criteria, 34 studies were eligible for this literature review [1,6,7,8,9,10,11,12,13,14,15,16,17,18,19,20,21,22,23,24,25,26,27,28,29,30,31,32,33,34,35,36,37], most of which (*n* = 22/34, 64.7%) included only one case or a series of fewer than five cases. The final entire sample included 149 patients, whose data are separately reported in Table 1 and Table 2 and summarized in Table 3 and Table 4.

### 3.1. Demographic, Clinical, Neuroradiological and Pathological Data (Table 1 and Table 3, Figure 2)

The overall sample included 99 (66.9%) females and 49 males (33.1%), with a median age of 54.88 years (range 22–79 y.o.). Presenting symptoms were reported in 56.3% of cases and were mainly represented by nasal obstruction (*n* = 68/84, 81%), followed by facial discomfort (*n* = 37/84, 44%)—including facial pain and/or pressure—epistaxis (*n* = 13/84, 15.5%) and ocular impairment (*n* = 12/84, 14.3%)—including diplopia, epiphora, and gaze restriction.

Data on the site of origin of the lesion and its pattern of growth were reported in 96% of cases (*n* = 143/149). The most frequent site of origin was the ethmoid sinus (*n* = 97/143, 67.8%), followed by the nasal cavity (*n* = 65/143, 45.4%), frontal sinus (*n* = 34/143, 23.7%), maxillary sinus (*n* = 18/143, 12.6%) and sphenoid sinus (*n* = 5/143, 3.5%). From the site of origin, the lesion extended to the skull base (mainly anterior cranial fossa) in 24.5% of the cases (*n* = 35/143), whereas the orbital invasion was reported in 28.7% of the cases (*n* = 41/143), and mainly occurred through the lamina papyracea.

The diagnosis of biphenotypic sinonasal sarcoma was achieved through an immunohistochemical study in 37.6% of cases (*n* = 56/149), and with the integration of biomolecular examination in the remaining 62.4% (*n* = 93/149).

### 3.2. Treatment and Outcome Data (Table 2 and Table 4, Figure 2)

The time lap from clinical symptoms and/or signs onset to treatment is reported just in 11 out of 149 patients (7.3%) and it is 12 months (mean time).

In 104 cases (69.8%), the type of treatment adopted was described. Surgery was performed in all but five cases, where only a biopsy (4.8%) was carried out, and two cases (1.9%) where only the combination of radio- and chemotherapy was administered. In detail, surgical procedure was adopted as a unique treatment in 69 patients (66.3%), while it was followed by adjuvant radiotherapy in 20 cases (19.2%), by chemotherapy in 3 (2.9%), and associated with both radio- and chemotherapy in 5 (4.8%).

The description of the type of surgical approach selected was reported in 58 out of 149 cases (39%). The most adopted surgical option was the endoscopic endonasal route (*n* = 33/58, 56.9%), followed by the combined microsurgical transcranial–endoscopic endonasal approach (*n* = 17/58, 29.3%) and isolated microsurgical transcranial approach (*n* = 7/58, 12%). A transorbital approach was chosen in only one case (1.7%).

The extent of tumor resection was reported in 62 cases of the overall series (41.6%). It was gross total (GTR) in 49 (79%) and sub-total (STR) in the remaining 13 (21%).

Data concerning peri-operative complications were reported in only twelve cases (8%): they occurred in four patients (33.3%) and mainly consisted of transient CSF leak.

In regard to the outcome data, post-treatment clinical conditions were reported just in five patients, registering an improvement in all but one of them, where they remained stable.

Data on the recurrence rate were reported in 84 out of 149 patients (56.3%) during a follow-up from 1 to 9 years. Among them, local recurrence was observed in 22 cases (26.2%), including five patients who experienced more than one recurrence over their lifetime.

The status of 85 out of 149 patients of the overall series (57%) was reported at last follow-up (mean 4.6 years): seventy-seven of them (90.6%) were alive and eight died. Three died due to tumor persistence/progression, one due to surgical complications and four from other causes.

## 4. Discussion

BSNS exhibits unique characteristics, differing histologically from malignant sarcomas or other sinonasal cancers, harboring biphenotypic markers’ expression and a peculiar identity combining clinical, morphological, histological and genetic features [30].

Several neoplastic diseases and with a different grade of malignancy can affect the sinonasal region [38]; among them, biphenotypic sarcoma, albeit exceptional for incidence (1–5% of head and neck malignancies [13]), with only 149 cases identified in the present literature review, should be considered in the differential diagnosis. This lesion mainly affects female (ratio F:M = 2:1) and middle-aged populations; nevertheless, no reactivity was reported for sexual tumor markers such as estrogen and progesterone receptors.

As the tumor arises from the mucosa of the upper airway (nasal cavity, 45.4%) and/or air-filled cavities like paranasal sinuses (ethmoid, frontal, maxillary or sphenoid sinus, in 67.8%, 23.7%, 12.6% and 3.5%, respectively), the clinical onset is mainly represented by nasal obstruction (81%), followed by facial discomfort (44%), which are not specific clinical symptoms and very commonly shared with several different diseases, thus requiring a wide differential diagnosis often being underestimated.

BSNS presents some peculiar intrinsic features with intermediate biological behavior between malignant and benign tumors:

1. Slow growth: mean time from clinical onset to treatment is 12 months (even if cases up to 3 years are described (Table 2)); nevertheless, considering the small sizes and the function of the common sites of origin of the tumor—such as nasal cavity and paranasal sinuses—as well as of the adjacent structures usually involved—such as the orbit—it is easy to understand that a tumor becomes symptomatic quite early;
2. Local aggressiveness: the tumor invades and destructs adjacent structures, both bony and soft tissues, including the medial wall, floor and roof of the orbit, cribriform plate, and orbital fat; therefore, a prompt and proper diagnosis and treatment are mandatory to prevent neuro-ophthalmological complications, such as CSF leak, meningitis, meningocele, seizures, pneumocephalus, anosmia, proptosis, and diplopia;
3. Infiltrative pattern of growth: this makes it hard to achieve clear margins after surgical excision despite the high rates of gross total resection and low rate of peri- and post-operative complications;
4. Long time to and very low frequency of malignant transformation;
5. Tendency to locally recur: Recurrence was observed in patients regardless of the extent of tumor resection and the administration of adjuvant radiotherapy; there is no significant evidence to support the need for concomitant radiotherapy or surgical excision alone. Post-operative RT is mainly adopted when the examination of the surgical margins is found to be positive or inconclusive [6]. Therefore, it is important to collect further studies with large case series and long follow-up to analyze the main risk factors for recurrence.
6. No distant metastasis.

BSNS is a primarily local aggressive disease, with the involvement of highly functional anatomical structures such as the upper respiratory tract, the orbit and the skull base; therefore, surgical resection represents the gold standard of treatment.

Nevertheless, due to the rarity of the pathology, well-defined guidelines of treatment, as well as a surveillance protocol, are missing, and management varies among different institutions, with most centers proposing the surgical procedure as the best option, both as a unique treatment and combined with radio- or chemotherapy, or radio- and chemotherapy.

In regard to the surgical strategy, including the goal of surgery and the approach selection, several factors, both related to the patient and pathology, must be considered.

In the presence of young patients with good clinical conditions and long expectancy of life, we consider that the maximal safe tumor resection should be attempted. Conversely, in the presence of elderly patients, with not a long expectancy of life, unnecessary overtreatment should be avoided and other primary goals should be pursued, including (1) subtotal resection through tumor debulking to ensure the patency of the airways and drainage of the affected paranasal sinuses; (2) resolution of the mass effect on the adjacent structures to stop and/or prevent a further worsening of neuro-ophthalmological deficits; and (3) preventing associated intracranial complications, like mucocele, CSF leak, pneumocephalus, meningitis, seizure, brain abscess, and subdural empyema. The extended endoscopic endonasal approach (EEEA) plays the leading role among surgical procedures for addressing pathologies of the ventral midline skull base [39,40,41,42,43,44]; in fact, it represents the most adopted surgical option for BSNS, isolated or in a combined manner with the transcranial approach. As an alternative, a microsurgical transcranial approach (TCA) is reserved for cases with large intracranial extension due to skull base invasion and it not being suitable for the EEEA. Finally, the transorbital approach (TOA) was selected just once. This option can be adopted in a combined bi-portal approach with the EEEA for BSNS with lateral extension to the paramedian and lateral aspects of anterior cranial fossa and orbital cavity [37]. Particularly, the endoscopic transorbital approach, initially mainly adopted by ophthalmologists for the management of intraorbital pathologies, owing to its peculiar advantages, over the last fifteen years, has become very popular among neurosurgeons for addressing lesions involving the paramedian regions of the anterior and middle skull base [45,46,47,48,49,50,51,52,53,54,55,56,57], and with spheno-orbital meningiomas, representing the optimal indication in carefully selected cases [58,59,60,61]. The combined endoscopic endonasal and transorbital approach for frontal sinus lesions is widely demonstrated as safe and effective [62,63,64]. In a recent paper, our group proposed a modular system of approach selection, considering the endoscopic endonasal route as the master approach and the endoscopic transorbital and open transcranial as complementary routes, which can be variously combined based on the tumor origin and pattern of growth [37]. In detail, the tumor component involving the midline structures, like the nasal cavity, ethmoid and frontal sinuses, can be addressed through the endoscopic endonasal approach; the tumor extension into the superolateral compartment of the orbit and/or the far lateral end of the frontal sinus can be approached through the endoscopic transorbital route; finally, for large intracranial tumor extension or involvement of the lateral end of frontal sinus bilaterally, a transcranial approach can be considered.

Despite the multicompartmental and locally invasive pattern of growth of BSNS, gross total tumor resection is achieved in 79% of cases, while a sub-total resection, including biopsies, is achieved in the remaining 21%. This accounts for the resolution of clinical symptoms and signs related to the mass-effect, which occurs in 80% of the cases.

Peri-operative complications are reported in four cases and mainly consist of transient cerebrospinal fluid leakage. This finding is understandable considering the aggressive and destructive nature of the lesion on the anterior skull base, especially on the cribriform plate, and the prevalent selection of the endoscopic endonasal route as a surgical approach, whose main complication is CSF leak [65,66,67,68]. Nevertheless, it should be considered that the continued refinements in skull base reconstruction techniques after endoscopic endonasal surgery [65,69,70] accounted for a decrease in the CSF leak rate under 5%.

At last follow-up, most patients were alive with no evidence of disease; among the eight cases of death reported, four (50%) were due to other causes, one was related to the surgical procedure and three we due to to tumor persistence/progression. Three cases of histologically tumor progression from low-grade to high-grade sarcoma were reported [20,21,25], of whom one died due to tumor progression, one died due to heart failure and one is alive.

### Limitations and Advantages of This Study

Its retrospective nature represents the first limitation of this study. In addition, the small size of the sample of patients included and the heterogeneity of the data represent other limitations of this study. Much of the data are incomplete, as such as those related to time to treatment, peri- and post-operative complications, clinical outcome, overall survival rate, and follow-up. Nevertheless, the present review is comprehensive and analyzes the main factors affecting the course of this rare disease, providing a significant contribution to better understand the natural history of BSNS and the impact of the different strategies of treatment on the outcome.

## 5. Conclusions

Biphenotypic sinonasal sarcoma is a rare and unique tumor entity in terms of biological and clinical behavior. Based on the current knowledge, surgery plays the leading role in treatment, accounting for gross total tumor resection in most cases, with clinical symptom and sign resolution and a low rate of peri-operative complications. The type of approach and the aim of surgery should be assessed case by case according to patient and pathology features. The role of adjuvant therapies is still unclear.

Further studies including large surgical series and those with long follow-up are required to define prognostic factors and guidelines of treatment for this peculiar pathological entity.

## Figures and Tables

**Figure 2:**
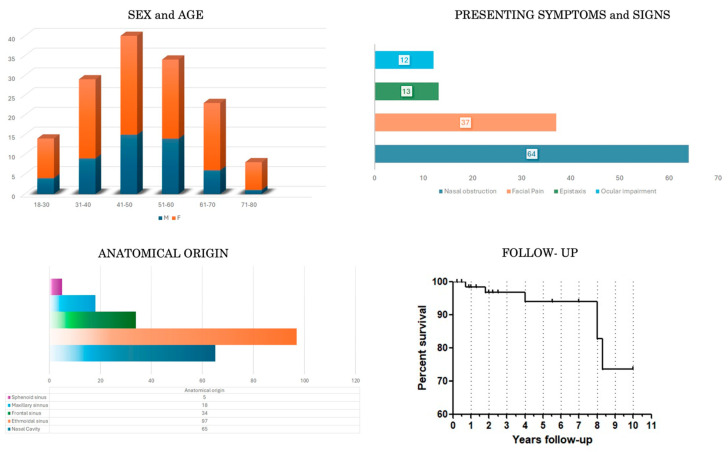
Histograms showing patient distributions for sex and age, main presenting symptoms and signs, anatomical origin of the lesion. Kaplan–Meier curve of the follow-up.

**Table 1:** Demographic, clinical, radiological and pathological data of 149 cases of BSNS.

Studies			Demographic and Clinical Data		Radiological Data			Diagnosis
	Authors/Year	Num of Cases	Sex, Mean Age (Years)	Presenting Symptoms	Anatomical Origin	Skull Base Involvement	Orbit Involvement	
1	Lewis et al. [1], 2012	28	21 F 7 M (52 years)	Breath difficulty, congestion, facial pressure	19 ES, 8 NC, 1 SS.	3 YES (ACF)	7 YES	Immunohistochemical
2	Powers et al. [7] 2015	1	M, 59	Sinusitis, congestion, facial pressure, anosmia, dysgeusia	ES-NC	YES (ACF)	None	Immunohistochemical
3	Rooper et al. [8] 2016	11	8 F 3 M (44 years)	n.a.	4 ES 3 FS 3 NC 1 ES-NC	None	2 YES	Immunohistochemical molecular
4	Wong et al. [9] 2016	1	M, 33	Recurrent brisk epistaxis	NC-SS	None	None	Immunohistochemical molecular
5	Huang et al. [10] 2016	7	4 M 3 F (52 years)	n.a.	2 FS 2 ES-NC 2 NC 1 ES	None	None	Immunohistochemical molecular
6	Cannon et al. [11] 2017	3	3 F (67.6 years)	Diplopia, facial discomfort, supraorbital swelling nasal obstruction, facial pressure	3 FS-ES	3 YES	3 Lamina papiracea	Immunohistochemical molecular
7	Lin et al. [12] 2017	1	F, 67	Nasal obstruction	ES-FS-SS-MS	YES	None	Immunohistochemical
8	Hockstein et al. [36] 2018	1	F, 79	Asymptomatic	FS	YES	Roof	Immunohistochemical
9	Andreasen et al. [13] 2018	3	2 F, 1 M (59.6 years)	Nasal obstruction and midfacial pressure	2 ES 1 ES-NC	None	None	Immunohistochemical
10	Koszewski et al. [35] 2018	1	M, 53	Unilateral nasal obstruction and epiphora	NC	YES (ACF)	Lamina papiracea	Immunohistochemical
11	Kakkar et al. [14] 2018	6	5 F, 1 M (51 years)	Nasal obstruction	1 NC 1 NC, MS 1 NC, MS, ES 1 NC, MS, ES 1 NC, MS, ES, FS 1 NC, ES	1 YES	None	Immunohistochemical
12	Quadros et al. 2019	1	F, 55	Obstruction of the left nasal cavity	NC	None	None	Immunohistochemical
13	Chitguppi et al. [6] 2019	1	M, 53	n.a.	ES-NC	YES	YES	Immunohistochemical molecular
14	Alkhudher et al. [16] 2019	1	F, 35	Nasal obstruction, epistaxis	NC, MS, ES	None	Lamina papiracea	Immunohistochemical
15	Miglani et al. [34] 2019	5	4 F, 1 M (56 years)	n.a.	5 NC-ES	5 YES (ACF)	5 Lamina Papiracea	Immunohistochemical
16	Fudaba et al. [15] 2019	1	M, 70	Loss of consciousness and vomiting	ES	YES	None	Immunohistochemical molecular
17	Le Loarer et al. [17] 2019	41	16 M, 25 F (51 years)	n.a.	14 NC 11 ES 10 ES-FS 6 n.a.	4 YES	4 YES	Immunohistochemical molecular
18	Kuhn et al. [33] 2019	1	n.a.	Worsening nasal obstruction, rhinorrhea, left orbital pain, proptosis and blurry vision	NC-ES	YES (ACF)	Lamina papiracea	Immunohistochemical molecular
19	Okafor et al. [32] 2020	1	M, 54	Left-sided nasal airway obstruction and anosmia	NC-MS-ES-FS	YES (ACF)	Lamina papiracea	Immunohistochemical
20	Okuda et al. [31] 2020	1	F, 64	Nasal obstruction	NC-MS-ES pterygopalatine fossa	YES (MCF)	YES	Immunohistochemical
21	Sethi et al. [18] 2021	3	3 F (56 years)	Left-sided nasal congestion and headaches/right nasal obstruction/rhinorrhea and left-sided nasal congestion	3 ES-MS-FS-NC	1 YES (ACF)	2 YES	Immunohistochemical
22	Hanbazazh et al. [19] 2021	1	M, 50	Orbital pain and pressure, diplopia, blurred vision, lateral gaze restriction	ES	YES	Lamina papiracea	Immunohistochemical molecular
23	Bell et al. [20] 2022	1	M, 66	Swelling of left eyelid, vertical diplopia and purulent nasal discharge	NC	YES (ACF)	YES	Immunohistochemical molecular
24	Hasnie et al. [21] 2022	1	F, 72	Nasal obstruction, episodic epistaxis and facial pressure/headaches, decreased sense of smell	MS-ES-Bilateral FS-NC	YES (ACF)	Lamina papiracea	Immunohistochemical molecular
25	Turri-Zanoni et al. [22] 2022	15	3 M, 12 F (54 years)	14 nasal airway obstruction 9 epistaxis, 6 olfactory disfunction3 facial pain	13 ES 2 FS	None	None	Immunohistochemical molecular
26	Nichols et al. [23] 2023	1	M, 54	Persistent headaches, postnasal drip, thickened nasal secretions, and epistaxis after sneezing	ES-SS	None	None	Immunohistochemical molecular
27	Ingle et al. [24] 2023	1	F, 47	Swelling eyelid, proptosis	NC, FS, ES, MS	None	Lamina papiracea	Immunohistochemical
28	Meyer et al. [25] 2023	1	M, 67	Nasal congestion and epiphora, right-sided ocular proptosis	ES-MS-FS	None	YES	Immunohistochemical molecular
29	Kominsky et al. [26] 2023	2	2 M (65 years)	Bilateral nasal congestion and blurry vision	ES-NC-FS	2 YES	2 Lamina papiracea	Immunohistochemical molecular
30	Bhele et al. [27] 2023	1	F,22	Vision loss, headache, hyposmia, facial pressure	NC-ES-SS-MS	YES (ACF)	Lamina papiracea	Immunohistochemical
31	Viramontes et al. [28] 2023	1	F, 40	Progressive obstruction of the right nasal cavity,	NC	None	None	Immunohistochemical molecular
32	Muraoka et al. [29] 2023	1	F, 73	Purulent nasal discharge and dull pain in the left cheek area	NC-ES-FS	YES (ACF)	None	Immunohistochemical molecular
33	Anastasiadou et al. [30] 2023	3	3 F (43 years)	Exophthalmos, headaches	NC-MS	1 YES	2 YES	Immunohistochemical molecular
34	Corvino et al. [37] 2024	1	M, 46	l. proptosis, upward gaze restriction	FS-ES	YES (ACF)	Roof	Immunohistochemical

M: male, F: female, n.a.: not available; l: left; ACF: anterior cranial fossa; mo.: months; ES: ethmoid sinus: FS: frontal sinus; SS: sphenoid sinus; MS: maxillary sinus; NC: nasal cavity.

**Table 2:** Treatment and outcome data of 149 cases of BSNS.

Studies			Treatment Data					Outcome Data at Last Follow Up		
	Authors/Year	Num of Cases	Time to Treatment	Type of Treatment	Type of Surgical Approach	EOR	Peri-Post Operative Complications	Clinics	Recurrence	Status
1	Lewis et al. [1] 2012	28	n.a.	n.a.	n.a.	n.a.	n.a.	n.a.	7/16 (range 12–118 mo.)	(mean 8.3 years) 14 alive 2 dead due to other causes
2	Powers et al. [7] 2015	1	n.a.	S	EEA	GTR	CSF leak	n.a.	None	Alive 10 mo.
3	Rooper et al. [8] 2016	11	n.a.	n.a.	n.a.	n.a.	n.a.	n.a.	2/7 (range 1–26 mo.)	(mean 4 years) 1/7 dead due to tumor
4	Wong et al. [9] 2016	1	n.a.	S Ad-CHT-RT	EEA	GTR	n.a.	n.a.	None	Alive 5 mo.
5	Huang et al. [10] 2016	7	n.a.	6 S 1 S + Ad-CHT-RT	n.a.	4 GTR	n.a.	n.a.	1/4 (36 mo)	(mean 8 years) 4 alive
6	Cannon et al. [11] 2017	3	n.a	2 S 1 Biopsy	1 EEA − 1 EEA + TCA1 EEA Biopsy	2 GTR 1 STR	n.a.	n.a.	1/3 (17 mo.)	(mean 25 mo.) 3 alive
7	Lin et al. [12] 2017	1	n.a	S	EEA	GTR	Subarachnoid hemorrhage; brain herniation	n.a.	n.a.	Dead due to surgery
8	Hockstein et al. [36] 2018	1	12 mo.	S	EEA + TCA	GTR	n.a.	n.a.	None	Alive
9	Andreasen et al. [13] 2018	3	n.a	1S 2 S + Ad.RT	n.a.	3 GTR	None	n.a.	1/3 (11, 21 and 24 mo)	(mean 67.3 mo.) 3 alive
10	Koszewski et al. [35] 2018	1	4 mo.	S + Ad.RT	n.a.	STR	n.a.	n.a.	None	Alive
11	Kakkar et al. [14] 2018	6	n.a.	3 S 3 Biopsy	3 EEA	4 STR	n.a.	n.a.	None	1/6 dead due to other causes
12	Quadros et al. 2019	1	n.a	S	EEA	n.a.	n.a.	n.a.	n.a.	n.a.
13	Chitguppi et al. [6] 2019	1	n.a.	S + Ad-RT	TCA + ETOA	STR	n.a.	n.a.	None	Alive
14	Alkhudher et al. [16] 2019	1	2 mo.	S	EEA	GTR	n.a.	Improved	None	Alive 2 years
15	Miglani et al. [34] 2019	5	n.a	4 S 1 S + Ad-RT	3 TCA 2 EEA	4 GTR 1 STR	n.a.	n.a.	2/5 (mean 31.4 mo.)	(mean 31.4 mo.) 5 alive
16	Fudaba et al. [15] 2019	1	REC after 11 years	S	EEA + TCA	GTR	n.a.	n.a.	No further	Alive
17	Le Loarer et al. [17] 2019	41	n.a.	20 S 8 S + RT 2 S + RT + CHT 1 RT+ CHT 2 S + CHT	n.a.	n.a.	n.a.	n.a.	8/25 (range 9–95 mo.)	(mean 45 mo.)
18	Kuhn et al. [33] 2019	1	n.a	S	TCA	GTR	None	n.a.	n.a.	n.a.
19	Okafor et al. [32] 2020	1	5 mo.	2 S	EEA	1 STR 1 GTR	None	n.a.	n.a.	n.a.
20	Okuda et al. [31] 2020	1	REC after 2 mo.	S + Ad.CHT	TCA	GTR	None	n.a.	YES (after 2 mo)	Dead 8 mo., death due to tumor progression
21	Sethi et al. [18] 2021	3	n.a	1 S + Ad.RT 2 S	3 EEA	3 GTR	None	n.a.	None	2 alive (mean 22 mo)
22	Hanbazazh et al. [19] 2021	1	36 mo	1 Biopsy 1 S 1 S + Ad.RT	Biopsy EEA TO TCA	STR	None	Improved	None	Alive
23	Bell et al. [20] 2022	1	REC after 15 years	1 S + Ad.RT	TCA	GTR	None	Stable	No further	Alive 10 mo.
24	Hasnie et al. [21] 2022	1	24 mo.	S	EEA + TCA	GTR	Infection, pneumocephal	n.a.	None	Death due to other causes
25	Turri-Zanoni et al. [22] 2022	15	n.a	13 S 2S + RT	7 EEA 8 EEA + TCA	13 GTR2 STR	n.a.	n.a.	1/15 (after 35 and 47 mo)	(27.3 months) 15 alive
26	Nichols et al. [23] 2023	1	n.a	S	EEA	n.a.	n.a.	Improved	None	Alive 3 mo.
27	Ingle et al. [24] 2023	1	2 mo.	S	EEA + TCA	GTR	n.a.	n.a.	None	Alive 3 mo.
28	Meyer et al. [25] 2023	1	36 mo.	Biopsy, RT, CHT	EEA	Biopsy	n.a.	n.a.	Progression	Dead 15 mo., death due to tumor progression
29	Kominsky et al. [26], 2023	2	3 weeks (1)	2 S	2 EEA	2 GTR	n.a.	n.a.	None	2 alive (mean 13 mo.)
30	Bhele et al. [27] 2023	1	8 mo.	Biopsy, Neo-CHT, S, Ad-PB	TCA + EEA	STR	n.a.	n.a.	None	Alive, 10 mo.
31	Viramontes et al. [28], 2023	1	n.a	S	EEA	GTR	n.a.	n.a.	None	Alive, 16 mo.
32	Muraoka et al. [29] 2023	1	n.a	S	TCA + EEA	GTR	n.a.	n.a.	None	Alive
33	Anastasiadou et al. [30], 2023	3	n.a	1 S, 2 S + Ad.RT	3 EEA	3 GTR	1 CSF leak	n.a.	None	Alive 7 years
34	Corvino et al. [37] 2024	1	2 mo.	S	TCA + EEA	GTR	None	Improved	None	Alive, 10 mo.

available data; n.a.: not available; GTR: gross total resection; STR: sub-total resection; S: surgery; RT: radiotherapy; CHT: chemotherapy; Ad: adjuvant; TCA: transcranial approach; EEA: endoscopic endonasal approach.

**Table 3:** Summarized available demographic, clinical, neuroradiological and pathological data of 149 cases of biphenotypic sinonasal sarcoma.

Covariates	Overall Sample 149 (%)	Statistical Analysis (*p* Value)
Demographic and clinical data		
Sex -F -M	148/149 * (99.3%) 99/148 (66.9%) 49/148 (33.1%)	*p* = 0.6
Age range (median)	22–79 years (54.88 y.o.)	*p* = 0.04
Main presenting symptoms -Nasal obstruction -Facial pressure/pain/discomfort -Epistaxis -Ocular impairment	84/149 * (56.3%) 68/84 (81%) 37/84 (44%) 13/84 (15.5%) 12/84 (14.3%)	*p* = 0.46
Radiological data		
Anatomical Origin -NC -ES -FS -MS -SS	143/149 * (96%) 65/143 (45.4%) 97/143 (67.8%) 34/143 (23.7%) 18/143 (12.6%) 5/143 (3.5%)	*p* = 0.32
Skull Base involvement -Yes -Not	143/149 * (96%) 35/143 (24.5%) 108/143 (75.5%)	*p* = 0.22
Orbit involvement -Yes -Not	143/149 * (96%) 41/143 (28.7%) 102/143 (71.3%)	*p* = 0.26
Pathological Diagnosis		
Diagnostic method -immunohistochemical alone -immunohistochemical and molecular	149/149 * (100%) 56/149 (37.6%) 93/149 (62.4%)	*p* = 0.55

* available data.

**Table 4:** Summarized available treatment and outcome data of 149 cases of biphenotypic sinonasal sarcoma.

Covariates	Overall Sample 149 (%)	Statistical Analysis (*p* Value)
Treatment Data		
Time to treatment (mean in months)	11/149 (7.3%) 12 months	*p* = 0.11
Type of treatment -S -S + RT -Biopsy alone -S + CHT -S + RT + CHT -RT + CHT	104/149 * (69.8%) 69/104 (66.3%) 20/104 (19.2%) 5/104 (4.8%) 3/104 (2.9%) 5/104 (4.8%) 2/104 (1.9%)	*p* = 0.43
Type of surgical approach -EEA -TCA -TOA -Combined	58/149 * (39%) 33/58 (56.9%) 7/58 (12%) 1/58 (1.7%) 17/58 (29.3%)	*p* = 0.1
EOR -GTR -STR	62/149 * (41.6%) 49/62 (79%) 13/62 (21%)	*p* = 0.45
Peri- and post-operative complications -Yes -None	12/149 * (8%) 4/12 (33.3%) 8/12 (66.7%)	
Outcome		
Clinical -Improved -Stable -Worsened	5/149 * (3.3%) 4/5 (80%) 1/5 (20%) ---	
Recurrence -Yes -Not	84/149 * (56.3%) 22/84 (26.2%) 62/84 (73.8%)	*p* = 0.6
Status -Alive -Dead	85/149 * (57%) 77/85 (91.8%) 8/85 (8.2%)	*p* = 0.87
Follow-up	Mean 4.6 years	St. Dev = 3.05

* available data. S: surgery; RT: radiotherapy; CHT: chemotherapy; TCA: transcranial approach; EEA: endoscopic endonasal approach; TOA: transorbital approach; GTR: gross total resection; STR: sub-total resection.

## Data Availability

Data of the current original research are available from the corresponding author on reasonable request.
